# TLR2 Derangements Likely Play a Significant Role in the Inflammatory Response and Thrombosis in Patients with *Ph*(−) Classical Myeloproliferative Neoplasm

**DOI:** 10.1155/2024/1827127

**Published:** 2024-08-09

**Authors:** Jen Chin Wang, Guanfang Shi, Chi Chen, Ching Wong, Vladimir Gotlieb, Gardith Joseph, Kiron V Nair, Lakshmi Boyapati, Enayati Ladan, James T. Symanowski, Lishi Sun

**Affiliations:** ^1^ Division of Hematology/Oncology Brookdale University Hospital Medical Center, Brooklyn, NY, USA; ^2^ Department of Biostatistics and Data Sciences Levine Cancer Institute, Charlotte, NC, USA

## Abstract

We investigated the role of toll-like receptors (TLRs) in inflammatory pathways in Philadelphia chromosome-negative myeloproliferative neoplasms (*Ph*(−)MPNs). TLR2 expression was increased in ET, PV, and MPN (grouped as (PV + (ET) + MF)), whereas TLR4 was elevated only in MPN. TLR3, 7, and 9 were not elevated. Cultured monocyte-derived dendritic cells and plasma assays in TLR2-elevated patients were found to secrete more cytokines than those from TLR2-normal patients. These facts suggest that TLR2 is the major inflammatory pathways in MPN. We also measured S100A9 and reactive oxygen species (ROS), revealing increased S100A9 in PV, MF, and MPN, while ROS were only increased in MF. These data suggests that MPNs initially involve TLR2, with minor contributions from TLR4, and with S100A9, leading to ROS formation, JAK2 mutation, and progression to MF or leukemia. Furthermore, patients with JAK2 mutations or leukocytosis exhibited higher TLR2 expression. In leukocyte–platelet interactions, cells from MPN patients displayed a stronger response to a TLR2 agonist than TLR4 agonist. A TLR2 inhibitor (but not a TLR4 inhibitor) attenuated this response. Thrombosis incidence was higher in TLR2-elevated patients (29%) than in TLR2-normal patients (19%). These findings suggest that TLR2 likely contributes to thrombosis in MPN.

## 1. Introduction

Chronic inflammation and oxidative stress have long been regarded as the key characteristics of myeloproliferative neoplasms (MPNs) over the past decade [1]. Inflammatory cytokines such as interleukins IL-1*β*, IL-6, IL-8, IL-10, IL-11, IL-12, IL-15, IL-17, and IL-33, as well as chemokine (C-X-C motif) ligand 1 (CXCL1), CXCL4, tumor necrosis factor-*α* (TNF-*α*), and others have been found to be elevated in both mouse models and patient samples [2]. Additionally, increased reactive oxygen species (ROS) due to persistent inflammation in MPNs have been well-documented [3].

Allogeneic stem cell transplantation has shown promise in fully restoring hematopoiesis and reducing bone marrow fibrosis in myelofibrosis patients, suggesting that neoplastic clones drive the inflammatory response, as evidenced by the transplantation's curative effects [4, 5]. However, the occurrence of gastrointestinal stromal tumors (GIST) post *Helicobacter pylori* infection, enteropathy-associated T cell lymphoma, and adenocarcinomas in individuals with coeliac disease indicates that inflammation may be the primary factor leading to carcinogenesis [6]. This prompts an analogy of whether inflammation or abnormal clones serve as the initial triggers of MPNs, much like the classic chicken-or-egg dilemma [7].

The onset of the inflammatory cascade can be attributed to danger-associated proteins like S100A9 and S100A8 [8] or high mobility group box 1 protein (HMGB1) [9] along with pathogen-associated molecular patterns (PAMPs) [10]. These proteins activate toll-like receptors (TLRs) and subsequent pathways, leading to the generation of ROS [10]. The prolonged inflammation due to these triggers can result in the accumulation of more ROS, cellular DNA damage, and clonal proliferation, ultimately propelling disease progression toward full-fledged MPNs [11].

Therefore, we study TLRs, S100A9, and ROS in the inflammatory pathway in MPN to see if these pathways were altered and important in the pathogenesis of MPN.

## 2. Subjects, Materials, and Methods

### 2.1. Patients

One hundred nineteen patients with MPN diseases (15 PMF, eight post-PV-MF, eight post-ET-MF, 51 ET, and 37 PV) were enrolled in the study, along with 21 normal volunteer controls. The diagnoses were made according to the World Health Organization classification [12]. Patients treated with ruxolitinib or other immunomodulatory drugs (IMiDs) were excluded from the study. The clinical features of the patients are listed in Table 1. Informed consent was obtained from all patients for peripheral blood collection in accordance with the Declaration of Helsinki. The protocol was approved as protocol No. 16-09, in 2016, by the Institutional Review Board of Brookdale University Hospital Medical Center.

### 2.2. TLR Assay

Mononuclear cells (MNC) were isolated from peripheral blood (PB) by gradient centrifugation with Ficoll–Paque. Then, quantification of TLR2, TLR3, TLR4, TLR7, and TLR9 was performed by immunostaining 1 × 10^6^ MNC that were incubated with fluorescence-conjugated anti-TLR2, anti-TLR3, anti-TLR4, anti-TLR7, and anti-TLR9 antibodies, respectively, and assayed by flow cytometry. Fluorescence-conjugated anti-CD3, CD14, CD20, CD34, CD41, and CD71 antibodies (Miltenyi Biotec, Auburn, CA) were used to gate and identify different cell populations.

### 2.3. Inflammatory Cytokines

*Cytokine ELISA Assay*. The Meso Scale Discovery Multi-Spot Assay system was used for the multiplex ELISA assay. Human plasma and cell culture supernatants were analyzed in duplicates for 10 cytokines: IFN-*γ*, IL-1b, IL-2, IL-4, IL-6, IL-8, IL-10, IL-12p70, IL-13, and TNF-*α*.

### 2.4. HMGB1 Assay

For the HMGB1 assay, the HMGB1 ELISA kit from Immuno-Biological Laboratories, Inc. (IBL-America) was used. The plasma samples were diluted four times with the provided sample dilution buffer and assayed in duplicate according to the manufacturer's instructions.

### 2.5. RAGE (RT-PCR) Assay

Total RNA was extracted from mononuclear cells of normal controls or patients. Predesigned primers for RAGE and internal control genes were ordered from Qiagen (Germantown, MD). Real-time PCR was performed using SAdvanced™ Universal SYBR® Green Supermix (Bio-Rad, Hercules, CA) on a Bio-Rad iQ5 Multicolor Real-Time PCR Detection System. At least three housekeeping genes (ribosomal protein L4, TATA box binding protein, and tubulin-*α* 1b) were used as normalization controls. The expression of RAGE was compared with each internal control, and the average of three was used to calculate the ratio of the final patient to the normal.

### 2.6. Cell ROS Measurement

Cellular ROS was determined by a dichlorofluorescein (DCF) assay. PBMNCs (peripheral blood mononuclear cells) were incubated with 10 *μ*M 2,7-dichlorodihydrofluorescein diacetate (carboxy-H2DCFDA) (Life Technologies) for 30 min at 37°C. The oxidation of H2DCFDA was measured and analyzed by flow cytometry.

### 2.7. Platelet Activation

Whole blood from normal controls or patients was diluted in HEPES-buffered Tyrode's solution with 0.3% BSA. The diluted blood was incubated with platelet agonists PAM3CSK4 (TLR2 agonist) or lipopolysaccharide (LPS) (TLR4 agonist) for 15 min at room temperature in the presence or absence of TLR2 inhibitor C29 (InvivoGen, San Diego, CA) or TLR4 inhibitor C34 (Sigma–Aldrich, St. Louis, MO). Platelet activation markers (PAC-1, P-selectin, CD40L) were labeled with fluorescence-conjugated antibodies (Miltenyi Biotec, Auburn, CA) and assayed by flow cytometry. Using flow cytometry, the interaction between leukocytes and platelets (LPI) was identified by CD45 and CD41 antibodies (Miltenyi Biotec, Auburn, CA), and LPI was counted as a percentage of CD45 cells with aggregated CD41 cells.

### 2.8. Statistical Analyses

Statistical analyses were performed with paired or unpaired Student's *t*-test and one-way ANOVA for comparison of groups. Results are given as mean with standard error and 95% confidence intervals (CIs). Correlations of TLR with clinical parameters were made by comparing TLR levels in WBCs (>10.0 × 10^9^/l vs. <10 × 10^9^), platelet counts (>400 × 10^9^/l vs. <400 × 10^9^), HB (>13 g/dl vs. <13), and JAK2 status (positive for Jak2 V617F mutation vs. negative) using the Student's *t*-test. TLR2-elevated patients (TLR2-E) were defined as patients with TLR2 values more than (>310.4), calculated as more than 2 standard deviation of control values (220 + 2 × 45.2 = 310.4) (MFI). This study was not prospectively powered to identify a targeted sample size to detect prespecified effect size differences in TLR2 values between the controls versus ET, PV, or MF groups. Convenience sampling was used and resulted in sample sizes of controls = 21, ET = 51, PV = 37, and MF = 31.

## 3. Results

### 3.1. TLR2 Levels Were Elevated in PV and MPN (ET, PV, and MF Grouped as MPN)

**Figure 1:**
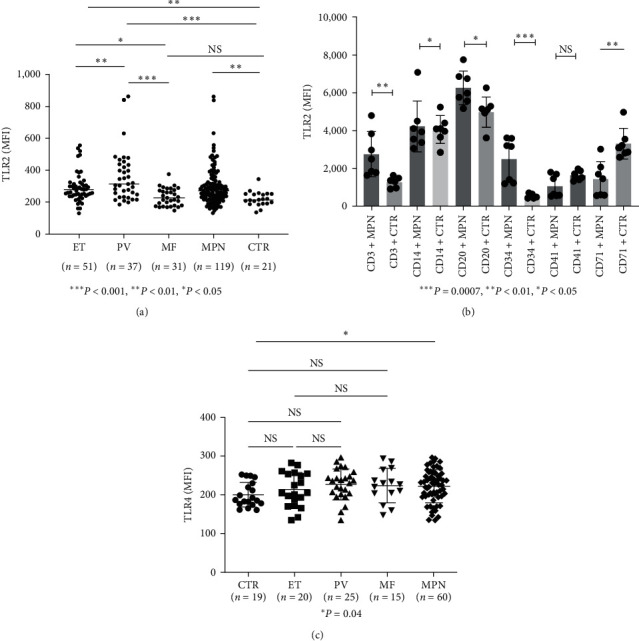
(a) Blood TLR2 levels were elevated in PV or MPN (ET, PV, and MF grouped as MPN). TLR2 levels were measured by flow cytometry (MFI). One hundred nineteen MPN patients (51 ET, 37 PV, 31MF (including eight post-ET MF, eight post-PV-MF, and 15 PMF)) were compared with 21 normal volunteer controls. The mean TLR2 values were ET (290.6 ± 9.8), PV (358.5 ± 26.01), MF (231.3 ± 9.89), and controls (220.6 ± 9.8). TLR2 was significantly elevated in the PV (*P*  < 0.001), ET (*P*  < 0.01), and MPN (grouping ET, PV, and MF together as MPN) (*P*  < 0.01) relative to the controls. MF was not significantly different from controls. Pairwise comparison the three MPN, ET was less than PV (*P*  < 0.01), and PV (*P*  < 0.001) and ET (*P*  < 0.05) were more than MF. (b) Patients with elevated TLR2 level (values more than 2 standard devation of controls) have increased TLR2 expression in CD34^+^, CD3^+^, CD14^+^, CD71^+^, and CD20^+^ cells; CD41^+^ cells were not over expressed. (c) TLR4 levels were not significantly elevated in PV, ET, or MF compared to controls but were significantly elevated when PV, ET, and MF were grouped as MPN. TLR4 was measured by flow cytometry (MFI). Sixty MPN patients were studied, including 20 ET, 25 PV, 15 MF, and 19 controls. The mean TLR4 values were ET (213.2 ± 9.8), PV (227.5 ± 7.9), MF (223.6 ± 11.2), and controls (200.1 ± 7.2). There were no significant differences when comparing the ET, PV, and MF groups to the controls, but when ET, PV, and MF were grouped as MPN, a significant difference was detected (*P*=0.04).

TLR2 levels are presented in Figure 1. One hundred nineteen MPN patients, including 51 ET, 37 PV, and 31 MF (which comprised eight post-ET MF, eight post-PV MF, and 15 PMF), were compared to 21 normal volunteer controls. The mean TLR2 values were as follows: ET (292.3 ± 13.13), PV (358.5 ± 26.01), MF (231.3 ± 9.89), and controls (220.6 ± 9.8). The overall comparison of TLR2 values among the four groups was highly significant (*P*  < 0.001). TLR2 was significantly elevated in PV (*P*  < 0.001), ET (*P*=0.009), and MPN (including ET, PV, and MF grouped as MPN) (*P*=0.004) compared to controls. Pairwise comparisons between the three MPN groups all show statistically significant differences. Mean ET was less than PV (*P* < 0.05) while ET was greater than MF (*P*=0.011). Additionally, PV TLR2 values were significantly greater than MF (*P*  < 0.001). These findings suggested PV and ET had higher TLR2 values, whereas MF patients had lower TLR2 values. For the post-ET MF or post-PV MF, none of patients were found to have elevated TLR2 values, and neither of patients with PMF has elevated values as well, so it is likely with elevated TLR2 in PV or ET, TLR2 values will become normal when they transformed to MF. There were no sequential TLR2 values before and after ET or PV transformation to MF. To investigate whether hydroxyurea treatment could influence TLR2 values in MPN patients, patients treated with hydroxyurea were compared to those who did not. The results showed no significant difference (*Supplementary figure 1*). We also further defined which cell populations that patients with Elevated TLR2, we found that TLR2 has increased expression in CD34^+^, CD3^+^, CD14^+^, CD71^+^, CD20^+^ cells, as shown in Figure 1(b). This suggests that TLR2 expression was increased in many cells including stem cells and immune cells.

### 3.2. TLR4, 3, 7, and 9

Sixty MPN patients were assayed for TLR4 levels, including 20 ET, 25 PV, 15 MF (comprising three post-ET MF, three post-PV MF, and nine PMF), and 19 controls. The mean ± SE (MFI) values were ET (213.2 ± 9.8), PV (227.5 ± 7.9), and MF (223.6 ± 11.2), compared to 200.1 ± 7.2 for the control group. There were no significant differences in ET, PV, or MF compared to controls. However, when ET, PV, and MF were grouped as MPN, they were elevated from the control group (*P*=0.04) (Figure 1(c)). TLR3, 7, and 9 did not demonstrate any significant difference among MPN patients with ET, PV, or MF or when grouped as MPN (*Supplementary figure 2*).

### 3.3. TLR2 in the Cytokine Production

**Figure 2:**
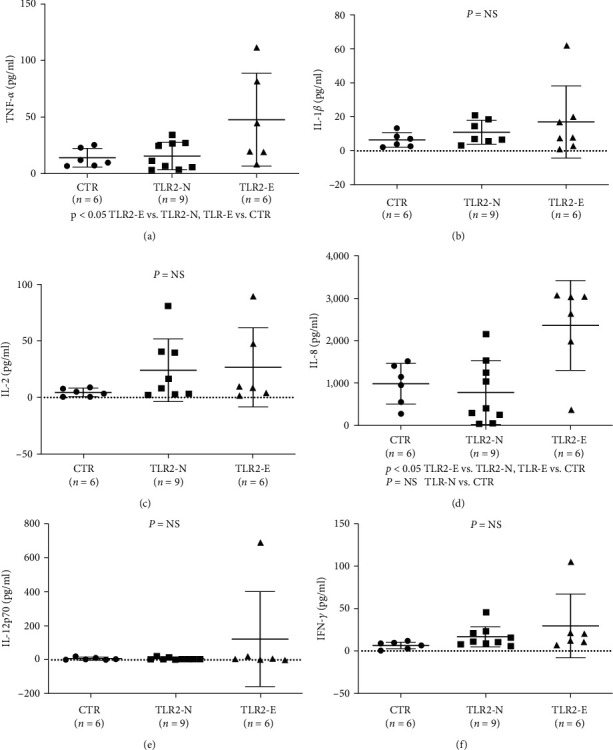
Monocyte-derived dendritic cells (mdDCs) and inflammatory cytokines in relation to blood TLR2 levels. mdDCs were generated from isolated monocytes (anti-CD14-coated magnetic beads) and then incubated with IL-4 and GM-CSF for 4 days. Immature mdDCs (1 × 10^4^/well) were either left untreated or stimulated with Pam3CSK4 in 96-well plates. After 48 hr, cells were incubated with 20% human AB serum in PBS containing 0.1% BSA and 0.1% NaN3 for 20 min at 4°C. After that, cells were stained with fluorescence-labeled mAb specific to CD80, CD83, CD86, and patients with elevated TLR2 values were compared with patients with normal TLR2 values on their mdDC production. There was no difference in the maturation of CD80 or CD 83 cells of mdDCs (data not shown). In TLR2-elevated patients, after stimulation with TLR2 ligand Pam3CSK4, there was significantly elevated production of TNF-*α* and IL-8 (a and d) and no difference in IL-1b, IL-2, IL-12p70, and IFN-*α* (b, c, e, and f).

**Figure 3:**
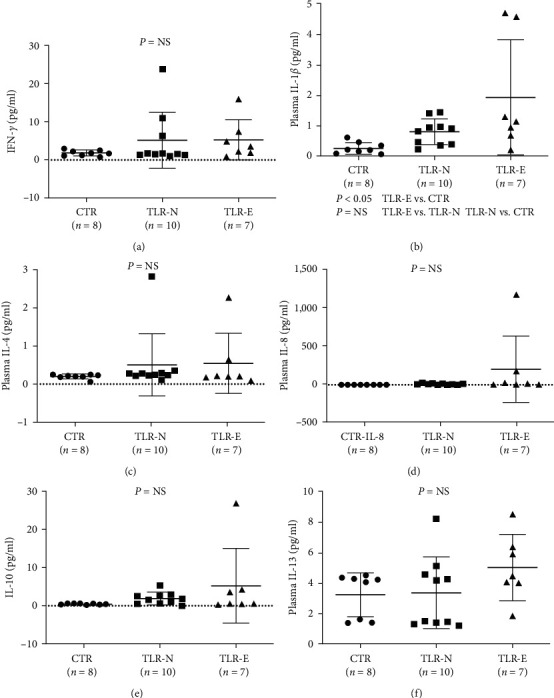
Plasma cytokine production in relation to blood TLR2 levels (a, b, c, d, e, and f). Multiple ELISA measured plasma cytokine. Plasma cytokine production was also compared in TLR2 elevated and normal TLR2 value patients. Plasma IL1*β* was elevated in TLR2-elevated patients (b), but there were no differences for IFN-*α*, IL-1b, IL-2, IL-4, IL-6, IL-10, IL-12p70, and IL-13 levels (a, c, d, e, and f).

Monocyte-derived dendritic cells (mdDC) and plasma inflammatory cytokines: To demonstrate that TLR2 is the primary innate immunity mediator, mdDCs were cultured from monocytes, as described. Patients with elevated TLR2 values were compared to those with normal TLR2 values regarding their mdDC production and cytokine production [13]. There was no difference in the maturation of mdDC to CD80 or CD83 cells (data not shown). However, TLR2-E patients demonstrated significantly higher production of TNF-*α* (Figure 2(a)) and IL-8 (Figure 2(d)), with no statistically significant difference, observed on IFN-*α*, IL-1b, IL-2, IL-4, IL-6, IL-10, IL-12p70, and IL-13 after stimulation with the TLR2 ligand Pam3CSK4 (Figures 2(b), 2(c), 2(e), and 2(f)). The lack of significant differences in IFN-*α*, IL-1b, IL-2, IL-4, IL-6, IL-10, IL-12p70, and IL-13 is likely due to the sample size, as all the cytokines appeared to be elevated in TLR-elevated patients than controls. Furthermore, plasma cytokine production was compared, and plasma IL1*β* was elevated in TLR2-E patients (Figure 3(b)), and other plasma cytokines were not significantly elevated (Figures 3(a), 3(c), 3(d), 3(e), and 3(f)). These findings suggest that TLR2 likely plays a significant role in inflammatory cytokines production which elevated cytokines were characteristically found in MPN patients [2].

### 3.4. TLR2 Is Related to Jak2 Mutation and Leukocytosis

**Figure 4:**
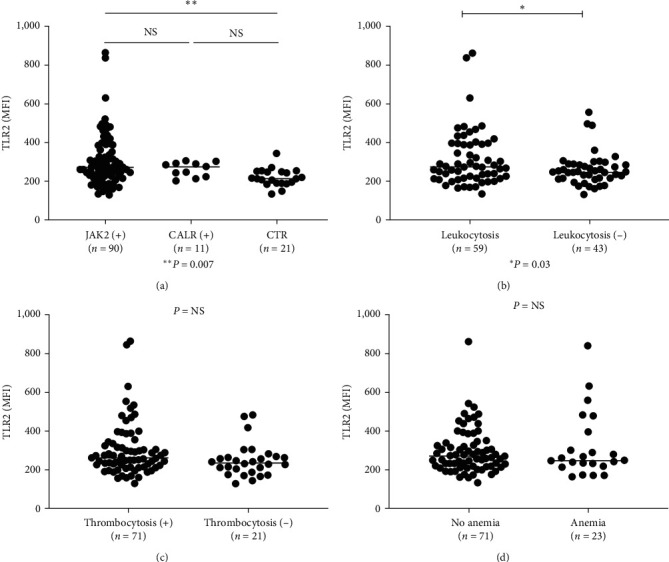
TLR2 values were significantly elevated in patients with JAK2 V617F mutations and leukocytosis but not in patients with thrombocytosis or anemia. (a) Ninety patients with JAK2 V617F mutations were compared with 11 CALR mutations (including six Type 1 and five Type 2) (six ET and five PMF) and 21 controls. The mean TLR2 values were JAK2 V617F (305.0 ± 13.7), CALR mutations (262.8 ± 11.3), and controls (220.6 ± 9.8). Patients with JAK2 mutations had significantly elevated TLR2 values compared to controls (*P*=0.007), while JAK2 mutations were not significantly different from CALR mutations, and CALR mutation patients were not significantly different from controls. (b) TLR2 levels were significantly higher in patients with leukocytosis (>10 × 10^9^/l) than in patients without leukocytosis (333.0 ± 22.97 (*n* = 46) vs. 269 ± 14.45 (*n* = 36) (*P*=0.03). (c) TLR2 values were not significantly different in patients with thrombocytosis (>400 × 10^12^/l) vs. those without thrombocytosis. The mean TLR2 values were in patients with thrombocytosis (304.5 ± 16.5) (*n* = 55) vs. patients without thrombocytosis (251.2 ± 16.3) (*n* = 26) (*P* = NS). (d) TLR2 values were not significantly different in patients with anemia (men < 14 gm/ml, women < 13 gm/ml) compared to those without anemia (294.1 ± 13.9) (*n* = 46); (317.4 ± 35.6) (*n* = 37), respectively.

To further investigate whether TLR2 values were related to Jak2 V617F mutation, leukocytosis, thrombocytosis, or anemia status, 90 patients with JAK2 V617F mutations were compared to 11 CALR mutations (including six Type 1 and five Type 2; six ET and five PMF), along with 21 controls (Figure 4(a)). The mean TLR2 values were as follows: JAK2 V617F (305.0 ± 13.7), CALR mutations (262.8 ± 11.3), and controls (220.6 ± 9.8). Patients with JAK2 mutations had significantly higher TLR2 values than controls (*P*=0.007), while JAK2 mutations were not significantly different from CALR mutations, and CALR mutation patients were not significantly different from controls. We also compared TLR2 values in MPN patients with JAK2 (+) versus JAK2 (−), also no significant difference were found (data not shown), this demonstrated that MPN patients have significant elevated TLR2 values than controls irrespective JAK2 status. One patient with MPL gene mutations had a high TLR2 value of 555.5. To evaluate TLR2 values with blood counts, patients on hydroxyurea and with low blood counts were excluded (because hydroxyurea will decrease the blood counts). TLR2 levels were significantly higher in patients with leukocytosis (>10 × 10^9^/l) than those without leukocytosis, with (333.0 ± 22.97 (*n* = 53) vs. 269 ± 14.45 (*n* = 43), (*P*=0.03), respectively (Figure 4(b)). TLR2 values did not show any significant difference in patients with anemia (men < 14 gm/ml, women <13 gm/ml) compared to those without anemia, with (294.1 ± 13.9) (*n* = 71) vs. (317.4 ± 35.6) (*n* = 21) respectively (Figure 4(d)). TLR2 values also did not show any significant difference in patients with thrombocytosis (>400 × 10^12^/l) compared to those without thrombocytosis (Figure 4(c)) with TLR2 values with thrombocytosis (304.5 ± 16.5) (*n* = 71) vs. without thrombocytosis (251.2 ± 16.3) (*n* = 21) (*P* = NS).

### 3.5. S100A9 Assay

**Figure 5:**
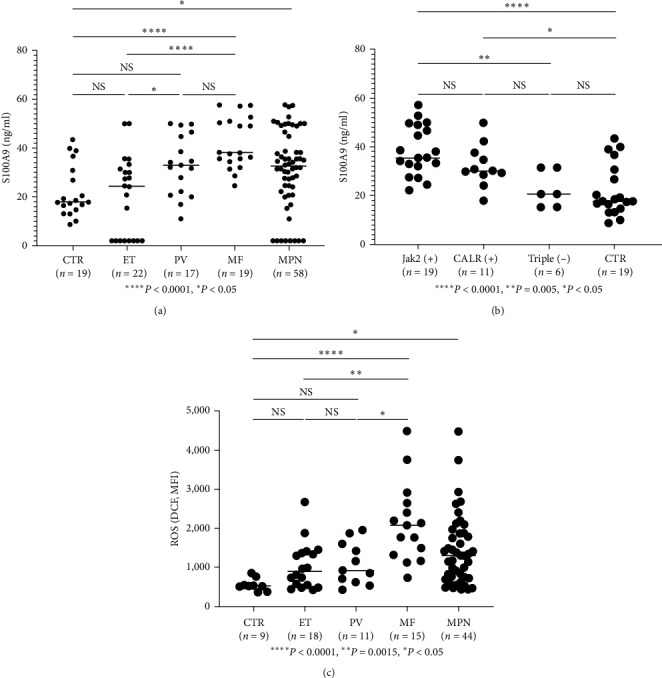
Plasma S100A9 levels in MPN. (a) Plasma S100A9 was measured in 58 patients with MPN (including 22 ET, 17 PV, 19 MF (4-ETMF, one 4-PVMF, and 11 PMF) and 10 controls. The S100 A9 results (ng/ml) were ET (20.57 ± 3.47) (*n* = 22), PV (33.20 ± 2.98) (*n* = 17), MF (42.02 ± 2.42) (*n* = 19), MPN (31.30 ± 2.11) (*n* = 58), and controls (22.17 ± 2.45) (*n* = 19). Plasma S100A9 was significantly elevated in PV (*P*  < 0.01), MF (*P*  < 0.0001), and MPN (*P*  < 0.05) compared with controls. MF was not significantly from PV but was significantly elevated relative to ET. PV was significantly elevated compared to ET, but the ET group was not different from the controls. (b) Jak2 mutant and CALR mutant patients also were found to have significantly increased than controls with mean ± SE of than controls (*P*  < 0.001), and CALR mutant patients were of 36.9 ± 1.7 (*P*  < 0.001) and 33.6 ± 2.5 (*P*=0.01) as compared to controls (22.5 ± 2.3), while there was no difference between JAK2 mutant and CALR mutant patients. (c) ROS in MPN. ROS was measured by flow cytometry in 44 patients with MPN, 15 MF (two patients with post-PV-MF, one with post-ET-MF, 13 patients with PMF), 18 patients with ET, and 11 patients with PV. The results (MFI, mean ± SE) show that ROS was elevated in the MF (2,127 ± 261.6, *n* = 15) group relative to the PV (1,090 ± 163.8, *n* = 11), ET (1,031 ± 140.9, *n* = 18), and controls (549.1 ± 52.50) (*n* = 9). MF was significantly elevated than PV or ET (*P*  < 0.01) and controls (*P*  < 0.001), while PV and ET were not significantly different from controls.

Plasma S100A9 was measured in 58 MPN patients, including 22 ET, 17 PV, and 19 MF (comprising 4-ETMF, one 4-PVMF, and 11 PMF) and 10 controls. The S100 A9 results (ng/ml) were as follows: ET (20.57 ± 3.47) (*n* = 22), PV (33.20 ± 2.98) (*n* = 17), MF (42.02 ± 2.42) (*n* = 19), MPN (31.30 ± 2.11) (*n* = 58), and controls (22.17 ± 2.45) (*n* = 19) (Figure 5(a)). Plasma S100A9 was significantly elevated in PV (*P*  < 0.01), MF (*P*  < 0.0001), and MPN (*P*  < 0.05) compared to controls. MF was not significantly different from PV but was significantly elevated compared to ET. PV was significantly higher than ET, but the ET group was not different from the controls. Moreover, Jak2 mutant and CALR mutant patients were also found to have significantly higher plasma S100A9 levels than controls, with mean ± SE of 37.1 ± 1.7 (*P*  < 0.001) and 33.6 ± 2.5 (*P*=0.01), respectively, compared to controls (22.5 ± 2.3). There was no significant difference between JAK2 and CALR mutant patients (Figure 5(b)), we also compared S100A9 levels in MPN patients with JAK2 (+) versus Jak2 (−), and fined no difference between the two groups, while MPN as a whole was significantly higher than controls (data not shown). This suggests S100A9 were significantly higher in MPN patients regardless of their JAK2 status.

### 3.6. ROS Measurement

ROS was measured in 44 MPN patients, including 15 MF patients (two post-PV-MF, one post-ET-MF, and 13 PMF), 18 ET patients, and 11 PV patients. The results (MFI, mean ± SE) showed that ROS was elevated in the MF group (2,127 ± 261.6) (*n* = 15) relative to the PV group (1,090 ± 163.8) (*n* = 11), ET group (1,031 ± 140.9) (*n* = 18), and the controls (549.1 ± 52.50) (*n* = 9). MF was significantly elevated compared to PV (*P*  < 0.05) or ET (*P*  < 0.0015) and controls (*P*  < 0.0001), while PV and ET were not significantly different from controls (Figure 5(c)).

### 3.7. HMGB1 Levels Were Not Elevated in the Plasma

As shown in *Supplementary figure 3*, plasma HMGB1 levels were measured in 83 MPN patients, including 37 ET, 23 PV, and 23 MF (comprising five post-ETMF, six post-PV-MF, and 12 PMF), along with 29 controls. The mean ± SE (*µ*g/ml) values were as follows: ET (2,415 ± 114.6), PV (2,316 ± 239.1), MF (2,568 ± 249.9), MPN (2,430 ± 107.6), and controls (1,873 ± 163.1). No significant differences were found for ET, PV, and MF, or when ET, PV, and MF were grouped as MPN, compared to controls.

### 3.8. RAGE Assay

RT-PCR of peripheral mononuclear cells was assayed for RAGE. As shown in *Supplementary figure 3*, 33 MPN patients (including 15 ET, eight PV, and 10 MF, comprising two post-ETMF, one post-PVMF, and seven PMF) were compared to controls. The mean ± SE of RAGE values was as follows: ET (1.02 ± 0.03) (*n* = 15), PV (0.97 ± 0.03) (*n* = 8), MF (1.11 ± 0.05) (*n* = 10), MPN (1.03 ± 0.02) (*n* = 33), and 0.99 ± 0.03 for the controls. No statistical differences were detected when comparing ET, PV, MF, and MPN to the controls.

### 3.9. Platelet Activation and Platelet–Leukocytes Interaction (LPI)

**Figure 6:**
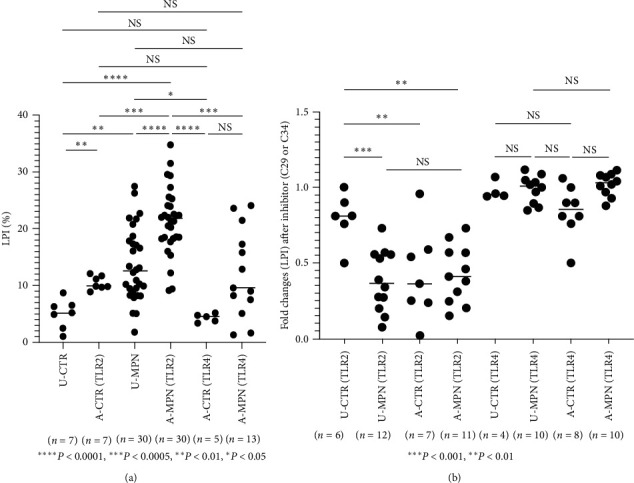
(a) Platelet activation and platelet–leukocytes interaction (LPI). Diluted blood was incubated with platelet agonist, PAM3CK4 (TLR2 agonist) or lipopolysaccharide (LPS) (TLR4 agonist), then the LPI was measured as the percentage of CD45 cells with aggregated CD41 cells. Thirty MPN patients including 16 ET, eight PV, and six MF (two post-PV-MF, one ET-MF, and four PMF) were compared to seven normal controls. The results showed that unactivated MPN (u-MPN) has more LPI than unactivated controls (u-CTR) with mean values of 13.01 ± 1.12 and 5.01 ± 0.97 in u-MPN and u-CTR, respectively (*P*  < 0.01). Upon activation by PM3CSK4, patients with MPN also have greater LPI than those by LPS (TLR4 agonist), with mean ± SE of 22.25 ± 0.99 and 10.46 ± 0.44, respectively (*P*  < 0.0005). (b) TLR Inhibitor in the leukocytes–platelet interactions (LPI). Diluted blood was incubated with platelet agonist, PAM3CK4 (TLR2 agonist) or lipopolysaccharide (LPS) (TLR4 agonist) for 15 min at room temperature in the presence or absence of TLR2 inhibitor C29 or TLR4 inhibitor C34. Fold changes were calculated as LPI after activation by either PM3CK4 (TLR2 agonist) or LPS (TLR4 agonist) or with an added inhibitor of C29 or C34, divided by the LPI before activation. Ten MPN patients were studied, including four ET, two ET-MF, one PV-MF, and two PV patients. The TLR2 inhibitor C29 significantly inhibits the LPI: expressed as (mean ± SE) of fold changes in unactivated MPN (0.38 ± 0.05), activated controls (0.42 ± 0.11), and activated MPN (0.42 ± 0.05). The TLR4 inhibitor C34 had no effects of inhibition: the unactivated MPN value was 0.99 ± 0.02, the activated MPN value was 1.02 ± 0.02, and the value of the activated control was 1.01 ± 0.02. These findings show TLR2 than TLR4 plays a significant role in the interaction of LPI in MPN patients.

Due to the significant association of TLR2 levels with Jak2 mutation and leukocytosis, which were reported to be associated with a higher incidence of thrombosis in MPN, studies were conducted to investigate platelet activation correlated with TLR2 levels. Aspirin slightly inhibits platelet activation, so patients taking aspirin were excluded from the study. Platelet activation markers, including PAC-1, P-selectin, and CD40L, did not consistently show results when whole blood was activated by PM3CSK4, as described in previous studies [14, 15]. The results were more consistent with the leukocyte–platelet interaction (LPI) response methods described in other studies [16, 17]. Therefore, to compare TLR2 and TLR4 in platelet activation, the LPI response method was used to assess the pre-thrombotic state. Sixteen ET, eight PV, and seven MF (including one post-PV-MF, one post-ET-MF, and five PMF) patients were compared to seven normal controls. The results (LPI), calculated as the percentage of CD45^+^ cells with aggregated platelet (CD41^+^), showed that unactivated MPN (u-MPN) patients had more LPI than unactivated controls (u-CTR), with mean ± SE of 13.01 ± 1.12 and 5.01 ± 0.97 in u-MPN and u-CTR, respectively (*P*  < 0.05). This is consistent with previous reports [16, 17] that unactivated MPN patients have more intrinsically activated platelets than unactivated controls (Figure 6(a)). Upon activation by PM3CSK4, patients with MPN also had greater LPI than controls, with a mean ± SE (LPI) of 22.25 ± 0.99 and 10.46 ± 0.44 in activated MPN and activated controls, respectively (*P*  < 0.01) (Figure 6(a)).

### 3.10. TLR2 than TLR4 Plays a Significant Role in the Leukocytes–Platelet Interactions

To investigate the potential roles of TLR2 and TLR4 in thrombosis in MPN, we observed that MPN patients activated by the TLR2 agonist (PM3CSK4) exhibited a significantly higher LPI response compared to activation by the TLR4 agonist (LPS), with mean ± SE values of 21.39 ± 1.07 and 12.12 ± 2.16, respectively (*P*  < 0.01). Subsequently, we experimented by adding the TLR2 inhibitor C29 (InvivoGen, San Diego, CA) or the TLR4 inhibitor C34 (Sigma–Aldrich, St. Louis, MO) to the LPI assay. The fold changes in LPI were calculated by dividing the LPI values after activation by either PM3CSK4 (TLR2 agonist) or LPS (TLR4 agonist), with or without added inhibitors, by the LPI value before activation. The study included 12 MPN patients, consisting of four ET, two ET-MF, one PV-MF, and five PV patients. As depicted in Figure 6(b), the TLR2 inhibitor C29 significantly suppressed LPI in unactivated MPN (0.38 ± 0.05), activated controls (0.42 ± 0.11), and activated MPN (0.42 ± 0.05). Conversely, the TLR4 inhibitor C34 had no inhibitory effect on unactivated MPN (0.98 ± 0.03), activated MPN (1.02 ± 0.02), or activated controls (0.84 ± 0.06) (Figure 6(b)). These results suggest that TLR2, rather than TLR4, plays a significant role in LPI in MPN patients. Consequently, TLR2 is likely to be important in thrombosis associated with MPN.

### 3.11. Comparison of the Thrombosis Incidence and Other Clinical Features in Patients with Elevated TLR2 and Normal Values

To further investigate the association between TLR2 and clinical thrombosis episodes, we compared patients with elevated TLR2 levels (defined as TLR2 values more than 2 standard deviations above the mean of normal controls, with values higher than 310.4 MFI) to patients with normal TLR2 levels.  Table 2 presents the findings, indicating that patients with elevated TLR2 levels show a slightly higher proportion of females and a greater prevalence of leukocytosis, with no significant differences in hemoglobin levels or platelet counts. Importantly, patients with elevated TLR2 levels experienced a higher incidence of thrombosis episodes, including both arterial and venous thrombosis (29%), compared to patients with normal TLR2 values (11%).

## 4. Discussion

TLR receptors play an important role as inflammatory cytokine receptors in the pathogenesis of MDS[18], and various other types of carcinoma [19]. In our studies on the inflammatory pathway in the pathogenesis of MPN diseases, we observed that TLR2 was significantly elevated in ET, PV, and MPN (grouped PV, ET, and MF) than controls (Figure 1(a)). TLR4 was elevated only when PV, ET, and MF were grouped as MPN (Figure 1(c)), while TLR3, 7, and 9 were not significantly elevated from controls *Supplementary figure 2* (since only 12 MPN patients were screened for TLR 3, 7, 9, an large cohort studies will be necessary to confirm these findings). These findings suggest that TLR2 is the major inflammatory pathway in *Ph*(−) MPN. In Figure 2, we further confirmed that in cultured dendritic cells (mdDC) from monocytes taken from patients with elevated TLR2 levels, more inflammatory cytokines, including IL-1*β* and IL-8, were secreted (other cytokines were found not to be significantly elevated, but they were shown to be increased compared to patients with normal TLR2 levels, suggesting that the lack of statistical significance was likely due to the sample size). Similarly, in the plasma from patients with elevated TLR2 levels, IL-1*β* was found to be significantly increased, while other cytokines were increased but not to a statistically significant degree (likely from sample size) (Figures 3(a), 3(c), 3(d), 3(e), and 3(f)). Therefore, these data support that TLR2, rather than other TLRs, plays a significant role in the inflammatory response in MPN.

S100A9 is predominantly expressed as a heterodimer together with S100A8[20]. S100A9 and S100A8 are essential in phagocytosis and play a crucial role in the pathogenesis of inflammatory disorders. Kovačić et al. [21] conducted an extensive study of S100 proteins in MPN diseases, which reported that S100A8/A9 were elevated in plasma and neutrophils of MPN disease and were correlated with the Jak2 mutation status. Our studies largely support their findings, indicating that S100A9 is elevated in MPN. Therefore, from our study and that of Kovačić, we can conclude that S100A9 likely plays a significant role in MPN diseases. Our previous finding that MDSCs were increased in MPN diseases [22] is also likely related, in part, to the elevated S100A9 and TLR2 levels in MPN, as S100A9 stimulates the formation of MDSCs [23].

HMGB1, besides acting as a pro-inflammatory DAMP, has also been observed to be overexpressed in several human tumor types, playing an important role in cancer progression and prognoses, such as acute myeloid leukemia [24] and other carcinomas [25]. RAGE serves as the receptor for HMGB1, and overexpression of RAGE and/or HMGB1 has been documented for various cancers [26]. Our studies did not detect a significant alteration of HMGB1 levels in plasma *Supplementary figure 3*. Additionally, we found that RAGE levels, as determined by RT-PCR of peripheral blood mononuclear cells *Supplementary figure 3*, did not significantly differ from controls. A large cohort study may be necessary to assess the role of HMGB-1 and RAGE in the inflammatory response and oncogenesis in MPN, or elevated HMGB1 may be increased intracellularly and cannot be detected in the plasma.

**Figure 7:**
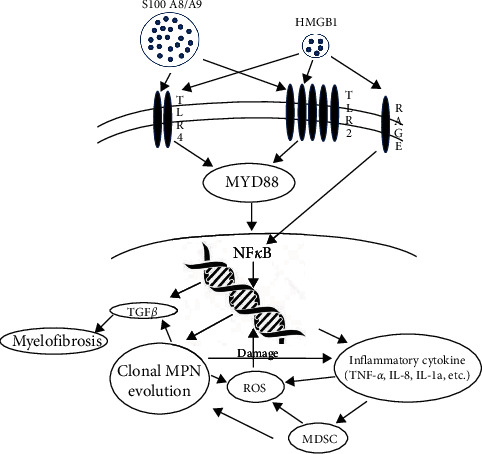
Summary of inflammatory pathway in the possible pathogenesis of MPN diseases. Initial inflammatory responses were largely mediated by TLR2 and less so by TLR4. Incorporating the S100A9/8 protein through MYD88 can induce NF-*κ*B formation, producing inflammatory cytokines, and producing ROS and MDSC formation. Years of the chronic inflammatory process and accumulation of ROS formation induce DNA damage, then clonal evolution, including JAK2, setting up a vicious cycle of JAK2 mutation refractory to cytokine inhibition, producing more ROS, and leading to the formation of other genetic marker gene mutations, thereby causing the formation of myelofibrosis or leukemia.

ET and PV eventually evolve into myelofibrosis, termed post-ET and post-PV MF, which have similar clinical features to primary myelofibrosis (PMF) [27, 28], although some authors found that post-ET and post-PV MF have a better prognosis than PMF [29, 30]. PMF, post-ET, and post-PV MF were found to have a higher JAK2 V617F allele burden[31] and genetic alteration marker changes, such as TET2, ASXL1, IDH, IKZF1, LNK, EZH2, DNMT3A, NF1, SUZ12, SF3B1, and SRSF2, than ET and PV [32]. This leads to the theory that MPNs are a disease process that progresses in a biological continuum from early cancer stages (ET/PV) to more advanced cancer stages (MF or acute myeloid leukemia) [33]. Our finding of increased TLR2 levels in PV, ET but not MF (Figure 1(a)), and markedly increased ROS production in patients with myelofibrosis compared to PV or ET (Figure 5(c)) could likely explain this observation. The initial enhanced inflammatory response by increased TLR2 in PV and ET results in the activation of NF-*κ*B and then ROS formation, leading to chronic inflammatory processes in MPN, with many years of accumulation and production of more ROS [33]. ROS induces more genetic instability and leads to the accumulation of genetic alterations, ultimately resulting in clonal evolution, such as JAK2 V617F [34]. This sets up a vicious cycle in which the poor negative response inhibition of the JAK2 mutation clone by inflammatory cytokine TNF-*α* [35] produces more ROS, inducing more genetic alteration changes that eventually lead to the development of myelofibrosis or leukemia, as proposed by Hasselbalch [36]. This may explain how PV and ET can develop myelofibrosis or leukemia after many years, as exemplified and summarized by the inflammatory pathways outlined in Figure 7.

The natural history of PV and ET is characterized by thrombo-hemorrhagic complications and a propensity for transformation into myelofibrosis (MF) and acute myeloid leukemia (AML). The 10-year risk of AML or MF in ET is less than 1% and 10%, respectively, while in PV, the 10-year projected rates of leukemic transformation and fibrotic progression are less than 5% and less than 10%, respectively [37, 38]. Thrombosis occurs in approximately one-third of PV patients and up to 29% of ET patients, but only in 13% of PMF patients, with a cumulative rate estimated to be around 3% per patient-year in PV and ET and approximately 2% per patient-year in PMF. Therefore, PV and ET demonstrate a higher incidence of thrombotic events than transformation to leukemia or MF, with both PV and ET carrying a greater risk of thrombosis than MF. The International Prognostic Score for Thrombosis in ET (IPSET-thrombosis) has identified age and a history of thrombosis as independent risk factors for future thrombotic events [39].

Other independent prothrombotic factors include cardiovascular (CV) risk factors and the JAK2V617F mutation [40], highlighting the independent thrombotic risk associated with the JAK2V617F mutation in MPN. Hobbs et al. [40] conducted a study using a JAK2V617F knock-in mouse model and observed that JAK2V617F-positive megakaryocytes displayed increased migratory ability and proplatelet formation, accompanied by enhanced platelet aggregation *in vitro* and thrombosis *in vivo*. Leukocytosis has also been associated with an increased thrombotic risk [41, 42], although certain meta-analyses have raised questions regarding these conclusions [43].

Furthermore, activated platelets play a central role in the prothrombotic state of MPN. Numerous studies have reported elevated platelet activation markers, such as P-selectin, CD40L, and platelet-leukocyte aggregates, in MPN, with some demonstrating a correlation between platelet activation and clinical thrombosis [44, 45]. Upon platelet activation, P-selectin and CD40 ligands are rapidly translocated to the platelet surface and subsequently cleaved to generate soluble, fully biologically active forms known as soluble P-selectin (sP-selectin) and soluble CD40 ligand (sCD40L). These soluble forms also promote coagulation by inducing tissue factor (TF) expression in monocytes and endothelial cells [44, 45].

TLRs are involved in platelet activation pathways through innate immune inflammatory responses or PAMP/DAMP ligation, leading to NF-*κ*B nuclear translocation and the production of pro-inflammatory cytokines, ultimately resulting in platelet activation [46, 47, 48]. Hence, TLR signaling likely plays a role in thrombotic pathways. Our data showed that *in vitro* platelet–leukocyte interaction (LPI) was enhanced by PM3CSK4 (a TLR2 agonist) but not by LPS (a TLR4 agonist) (Figure 6(a)). Furthermore, the enhanced LPI response was significantly diminished by a TLR2 inhibitor, but not by a TLR4 inhibitor (Figure 6(b)), indicating that TLR2 plays a major role in the platelet activation pathway in MPN. Additionally, our findings suggest that patients with elevated TLR2 levels experience a higher incidence of thrombotic episodes than patients with normal TLR2 levels (Table 2).

In conclusion, our study revealed the following: (1) Elevated TLR2 levels were observed in PV and ET patients compared to MF (Figure 1(a)); (2) TLR2 levels were associated with Jak2 mutations (Figure 4(a)) and leukocytosis (Figure 4(b)); (3) enhanced *in vitro* LPI was induced by PM3CSK4 (a TLR2 agonist), but not by LPS (a TLR4 agonist), and this response was markedly diminished by a TLR2 inhibitor, but not by a TLR4 inhibitor (Figures 6(a) and 6(b), clinical evidence showed that patients with elevated TLR2 levels experience more thrombosis than patients with normal TLR2 levels. Based on these findings, we conclude that TLR2 likely plays a major role in thrombosis in MPN. Further clinical studies correlating thrombosis incidence with TLR2 levels in a larger patient sample and extensive thrombosis panel investigations are necessary to confirm the significant involvement of TLR2 in thrombosis in MPN.

## Figures and Tables

**Table 1:** Characteristics of patients and controls.

	ET	PV	MF	Controls	*P* value
Age	61.3 ± 2.87 (*n* = 51)	70.8 ± 2.0 (*n* = 37)	67 ± 2.1 (*n* = 31)	61.2 ± 2.9 (*n* = 21)	*P* = NS, among ET, PV, MF, and controls

Gender, F (%)	57	63	23	50	—

HB (g/dl) (mean ± SE)	12.9 ± 0.70	14.6 ± 1.2	8.6 ± 0.8	13.5 ± 1.2	*P* = NS, ET vs. PV; *P* < 0.05, ET or PV vs. MF

WBC (×10^9^/l)	14.3 ± 3.1	14.7 ± 2.0	13.8 ± 2.1	7.5 ± 3.1	*P* = NS, among ET, PV, and MF

Platelet (×10^9^/l)	547.4 + 43.6	514.1 ± 45.2	594.1 ± 100.1	229.3 ± 59.1	*P* = NS, among ET, PV, and MF

**Table 2:** Clinical features of patients with TLE2-E compared to TLR2-N.

	TLR-E	TLR-N	*P* value
Age	70.4 ± 2.3	64.8 ± 1.7	NS
Female	54%	41%	—
Hb	13.3 ± 1.0	11.9 ± 0.50	NS
Platelet	543.6 ± 55.3	578.4 ± 61.8	NS
WBC	16.0 ± 1.8	11.5 ± 0.9	*P*=0.02
Thrombosis	7/24 (29%)	8/68 (11%)	—

TLR-E, patients with elevated TLR2 (defined as more than mean + 2 × SD of controls); TLR-N, patient with normal TLR2 levels.

## Data Availability

The data used to support the findings of this study are included in the article.

## Supplementary Materials

Supplementary 1 Figure S1: no significance of TLR2 levels in patients on hydroxyurea vs. those not on hydroxyurea. Thirty-two patients on hydroxyurea (including 13 ET, 10 PV, nine MF (four PV-MF, one ET-MF, four PMF)), and 29 patients not on hydroxyurea (including 15 PV, six ET, and eight MF (five PMF, one ET-MF, two PV-MF) were studied. We detected no statistical difference with TLR2 values (mean ± SE) in the hydroxyurea group (292.7 ± 16.1) and nonhydroxyurea group (333.5 ± 35.2).

Supplementary 2 Figure S2: no significant differences in TLR 3, 7, and 9. ET, PV, and MF groups (four patients each) were assayed for TLR3, TLR7, and TLR9 and compared with four normal volunteer controls. (A) The mean TLR3 values were ET (140.7 ± 15.07), PV (153.7 ± 10.0), MF (160.7 ± 11.50), MPN (151.7 ± 6.92) compared with controls (141.6 ± 18.75) (*P* = NS). (B) TLR7 is shown in Figure S1B, with mean values of ET (1,914 ± 85.46), PV (1,604 ± 166.7), MF (1,892 ± 67.89), and MPN (1,803 ± 73.65) (all *P*-values not significant relative to controls (1,588 ± 73.65)). (C) Mean TLR9 values: ET (6,540 ± 212.4), PV (6,127 ± 53.55), MF (6,314 ± 121.8), and MPN (6,327 ± 91.09) (all *P*-values not significant relative to controls (6,254 ± 267.2)).

Supplementary 3 Figure S3: (A) HMGB 1 levels are not elevated in the plasma (B) RT-PCR of RAGE. (A) Plasma HMGB1 levels were measured in 83 patients with MPN (including 37 ET, 23 PV, 23 MF (including five post-ETMF, six post-PV-MF, 12 PMF)) and 29 controls. There were no significant differences among different groups and controls. (B) RT-PCR of peripheral mononuclear cells was assayed for RAGE (the receptor for advanced glycation end-products). Thirty-three patients with MPN (15 ET, eight PV, 10 MF (two post-ETMF, one post-PVMF, seven PMF)) were compared to controls. The RAGE values were ET (1.02 ± 0.03), PV (0.97 ± 0.03), MF (1.11 ± 0.05), and MPN (1.03 ± 0.02) compared with controls (0.99 ± 0.03). No statistical difference between ET, PV, MF, or MPN relative to the controls.
