# Supplementary figures and images for: TLR2 Derangements Likely Play a Significant Role in the Inflammatory Response and Thrombosis in Patients with Ph(−) Classical Myeloproliferative Neoplasm
Source: Mediators Inflamm. 2024 Aug 9;2024:1827127. doi: 10.1155/2024/1827127 (PMC11329310; doi:10.1155/2024/1827127)

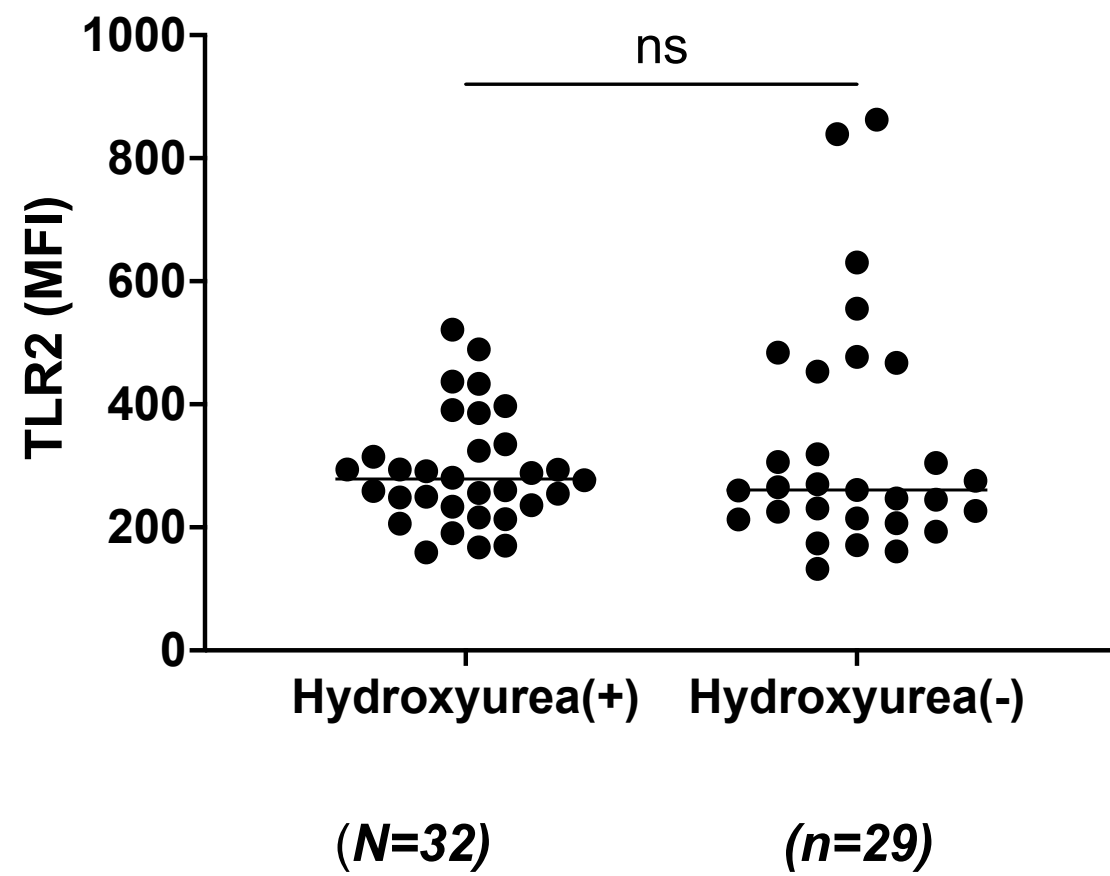

Supplement: Supplementary 1 — Figure S1: no significance of TLR2 levels in patients on hydroxyurea vs. those not on hydroxyurea. Thirty-two patients on hydroxyurea (including 13 ET, 10 PV, nine MF (four PV-MF, one ET-MF, four PMF)), and 29 patients not on hydroxyurea (including 15 PV, six ET, and eight MF (five PMF, one ET-MF, two PV-MF) were studied. We detected no statistical difference with TLR2 values (mean ± SE) in the hydroxyurea group (292.7 ± 16.1) and nonhydroxyurea group (333.5 ± 35.2). [file 1827127.f1.pdf]

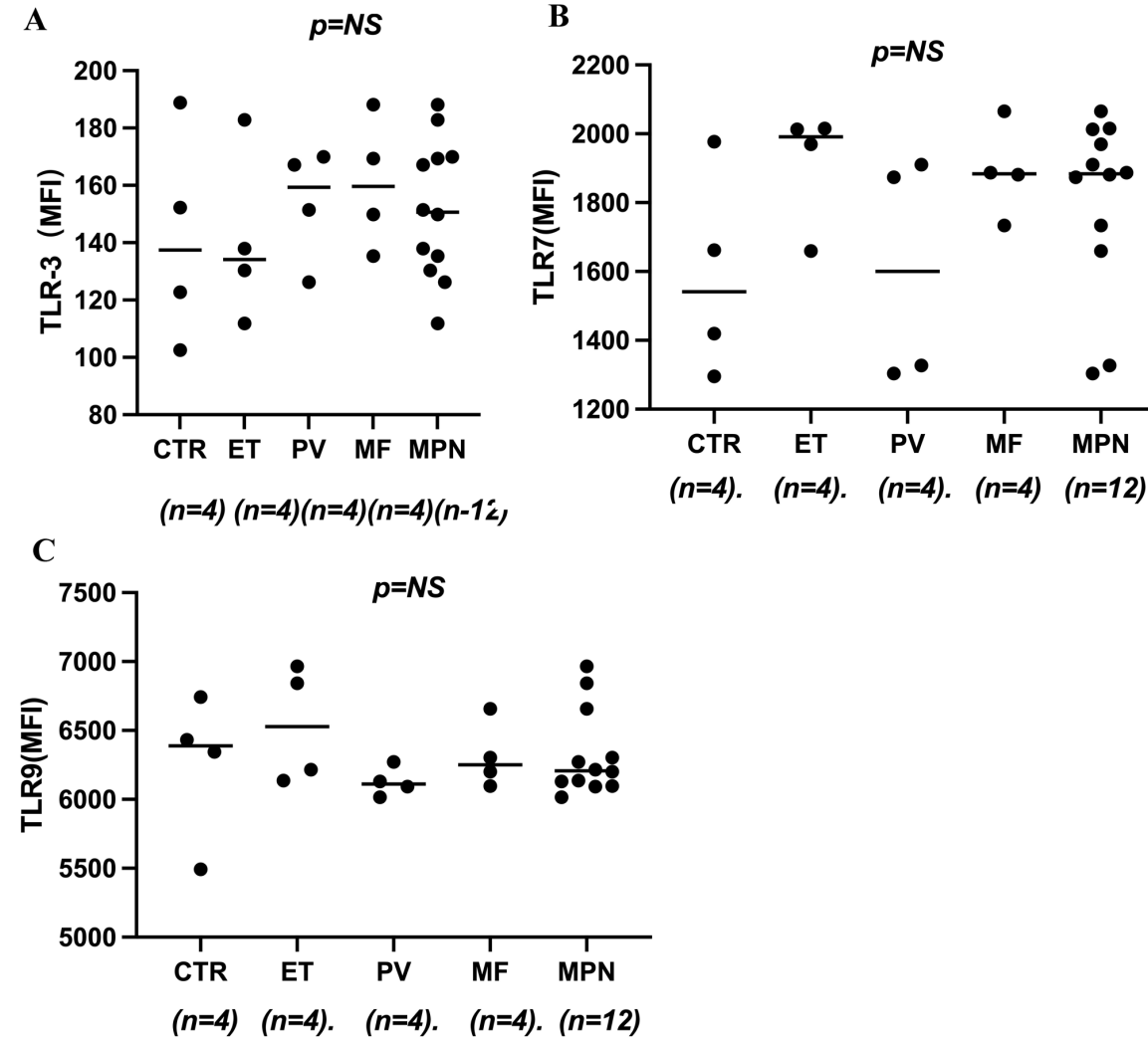

Supplement: Supplementary 2 — Figure S2: no significant differences in TLR 3, 7, and 9. ET, PV, and MF groups (four patients each) were assayed for TLR3, TLR7, and TLR9 and compared with four normal volunteer controls. (A) The mean TLR3 values were ET (140.7 ± 15.07), PV (153.7 ± 10.0), MF (160.7 ± 11.50), MPN (151.7 ± 6.92) compared with controls (141.6 ± 18.75) (P = NS). (B) TLR7 is shown in Figure S1B, with mean values of ET (1,914 ± 85.46), PV (1,604 ± 166.7), MF (1,892 ± 67.89), and MPN (1,803 ± 73.65) (all P-values not significant relative to controls (1,588 ± 73.65)). (C) Mean TLR9 values: ET (6,540 ± 212.4), PV (6,127 ± 53.55), MF (6,314 ± 121.8), and MPN (6,327 ± 91.09) (all P-values not significant relative to controls (6,254 ± 267.2)). [file 1827127.f2.pdf]

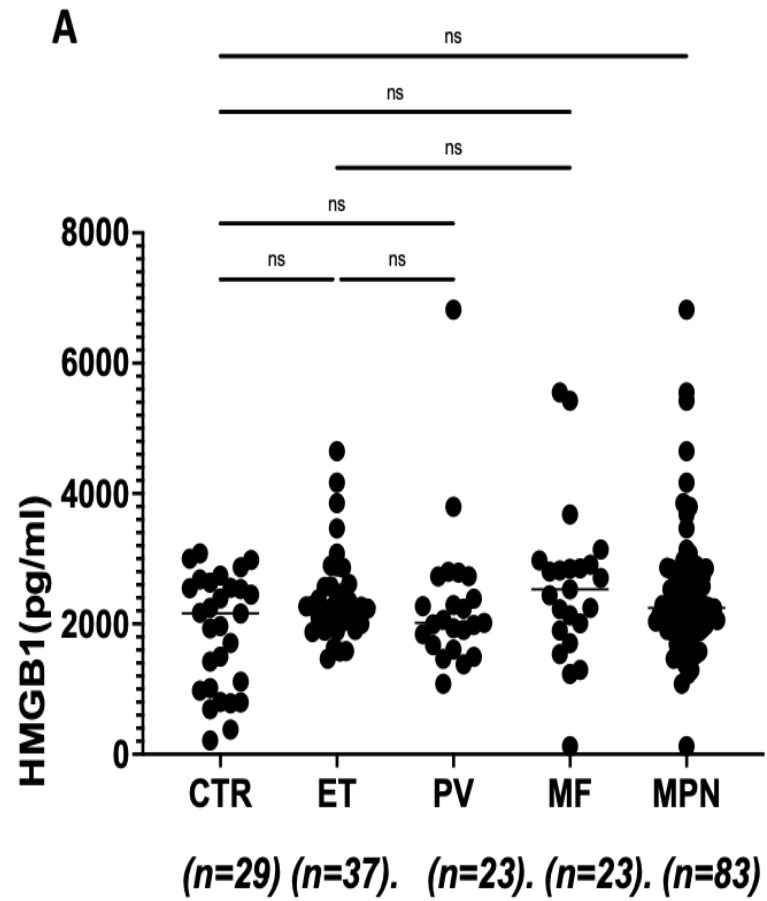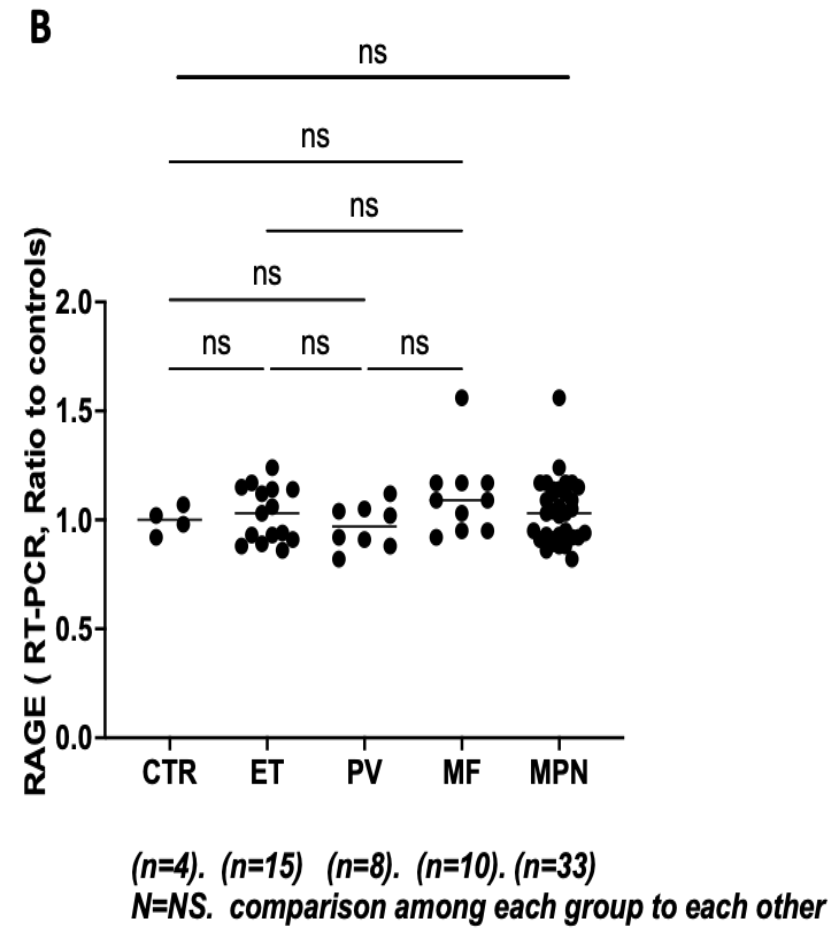

Supplement: Supplementary 3 — Figure S3: (A) HMGB 1 levels are not elevated in the plasma (B) RT-PCR of RAGE. (A) Plasma HMGB1 levels were measured in 83 patients with MPN (including 37 ET, 23 PV, 23 MF (including five post-ETMF, six post-PV-MF, 12 PMF)) and 29 controls. There were no significant differences among different groups and controls. (B) RT-PCR of peripheral mononuclear cells was assayed for RAGE (the receptor for advanced glycation end-products). Thirty-three patients with MPN (15 ET, eight PV, 10 MF (two post-ETMF, one post-PVMF, seven PMF)) were compared to controls. The RAGE values were ET (1.02 ± 0.03), PV (0.97 ± 0.03), MF (1.11 ± 0.05), and MPN (1.03 ± 0.02) compared with controls (0.99 ± 0.03). No statistical difference between ET, PV, MF, or MPN relative to the controls. [file 1827127.f3.pdf]
